# A potential bio‐control agent from baical skullcap root against listeriosis via the inhibition of sortase A and listeriolysin O

**DOI:** 10.1111/jcmm.14110

**Published:** 2018-12-25

**Authors:** Gejin Lu, Lei Xu, Tong Zhang, Xuming Deng, Jianfeng Wang

**Affiliations:** ^1^ Center of Infection and Immunity, First Hospital Jilin University Changchun Jilin, 130021 China; ^2^ Key Laboratory of Zoonosis, Ministry of Education Institute of Zoonosis, College of Veterinary Medicine, Jilin University Changchun Jilin, 130062 China

**Keywords:** anti‐virulence, baicalein, infection, *Listeria monocytogenes*, listeriolysin O, sortase A

## Abstract

*Listeria monocytogenes *(LM) is a classical model intracellular pathogen and the leading cause of listeriosis, which has long been a global public health issue. The successful infection of LM is related to a series of virulence factors, such as the transpeptidase enzyme sortase A (SrtA) and listeriolysin O (LLO), which are crucial for bacterial internalization and escape from phagosomes respectively. It is speculated that targeting multiple virulence factors may be due to a synergistic effect on listeriosis therapy. In this study, an active flavonoids component of *Scutellaria baicalensis *Georgi, baicalein, was found to potently block both listerial SrtA catalyzed activity and LLO hemolytic activity within 16 μg/mL. After pretreatment with baicalein, 86.30 (±11.35) % of LM failed to associate with Caco‐2 cells compared to the LM without preincubation (regarded as 100% internalization). Furthermore, baicalein addition may aid in bacterial degradation and clearance in macrophagocytes. During a 5 h observation, LM in cells incubated with baicalein showed significantly decreased vacuole escapes and sluggish endocellular growth. In addition, baicalein directly prevented LM‐induced cells injury and mice fatality (survival rate from 10.00% to 54.55% in 4 days post‐intraperitoneal injection). Taken together, as an antagonist against SrtA and LLO, baicalein can be further developed into a biotherapeutic agent for listeriosis.

## 1 INTRODUCTION

*Listeria monocytogenes* (LM) is a gram‐positive, facultative anaerobe first isolated in 1926, and a leading cause of severe infection with systemic disease, such as isolated bacteremia, maternal‐neonatal (MN) infection and human central nervous system (CNS) infection.^1^, ^2^ Immuno‐compromised individuals, the elderly, fetuses and newborns are high‐risk populations for listeriosis. In China, 253 invasive listeriosis cases were reported in 19 provinces from 2011 to 2016, and the total fatality rate of these cases was 25.7%.^3^ Other animals, such as birds and ruminants, which consumed contaminated feed, may also be infected by LM.^4^, ^5^

LM is an opportunistic pathogen and an important cause of human foodborne infection. Since the first antibiotic‐resistant LM strain was isolated from a patient in 1998, antibiotic resistance has become a public health concern during listeriosis therapy.^3^ These factors all demand for novel agents or strategies against LM infection. The inhibition of virulence factors has been proven to be a potent strategy to overcome infectious diseases.^6^, ^7^, ^8^ As LM is used widely as a model intracellular organism in infection biology and immunology, the study of listeriosis may be given to improve treatment of other gram‐positive and intracellular bacterial infections.^1^, ^9^

LM employs an intracellular lifecycle and is ingested by professional and non‐professional phagocytes (such as epithelial cells). The internalization of LM into epithelial cells is mainly based on the enzymatic activity of sortase A (SrtA). By using listeriolysin O (LLO), LM can lyse vacuoles and escape to reach the host cytosol and replicate. Finally, LM will move towards the membrane, form membrane protrusions and invade the adjacent cells.^10^ LM can assemble adherence and internalization‐related surface biotin to achieve internalization. The catalysing activity of SrtA, a kind of conservative cysteine transpeptidase enzyme widely existing in LM and other gram‐positive pathogens, is involved in the process of surface proteins covalently attaching onto bacterial peptidoglycan.^11^ SrtA is a target for anti‐listeriosis drugs, which has been investigated to contribute greatly to bacterial pathogenicity, as the mutation of SrtA resulted in a remarkably decreased virulence in cell models and in mouse models compared to wild type or single mutant internalin strains.^12^, ^13^, ^14^

Listeriolysin O (LLO), encoded by the *Listeria hly* gene, is a pore‐forming toxin which is called “the Swiss army knife of Listeria.^15^” LLO is a cholesterol‐dependent cytolysin (CDC) family member, is essential for lysing membrane‐bound compartments in a pH‐dependent manner and blocking phagosome‐lysosome fusion.^16^ Soluble LLO monomers bind to host membrane cholesterol, oligomerize and form large pores.^17^, ^18^ Strains with lower LLO expression failed to escape from phagosomes and had very slow replication in the internalization vacuole. However, *hly* mutants also displayed significantly attenuated phenotypes in in vivo infections.^18^

The root of *Scutellaria baicalensis* Georgi, Skullcap root, is a popular and multipurpose Chinese medicinal plant with a long use history in China and other East Asian countries as a therapeutic agent for fever, allergic reaction, or bacterial/viral infections.^19^, ^20^ As Skullcap root was traditionally utilized by oral administration, it is possible to discover a potential biocontrol agent against food‐born pathogen infection from Skullcap root exacts. Baicalein (5, 6, 7‐trihydroxyflavone) is one of the major flavone constituents, which was reported to attenuate inflammation and cerebral cortex apoptosis, and prevent γ‐induced neurogenesis impairment in recent researches.^21^, ^22^ The multifarious bioactivities of baicalein may be associated with the anti‐infectious mechanisms of Skullcap root.

In this study, we investigated the impact of baicalein on LM infection both in vitro and in vivo. We first determined the anti‐listerial SrtA peptidase activity of baicalein. The inhibition on SrtA significantly decreased colonization into epithelial cells. Simultaneously, baicalein decreased bacterial vacuole escape and multiplication owing to its efficient blockage of LLO. The inhibition of SrtA and LLO seems to be combined for cellular protection. Baicalein administration also was an effectual therapy in mouse models of peritoneal infection. Therefore, our results suggested that baicalein is a potential anti‐LM infection compound.

## 2 MATERIALS AND METHODS

### 2.1 Animals and cell line culture

In this study, 6‐ to 8‐week‐old female‐Balb/C mice (18‐20 g) were obtained from Liao Ning Chang Sheng Biotechnology Co., Ltd. and kept at a room temperature for at least 4 days before infection. The mice used for the experiments were housed and handled in accordance with the guidelines established by the United States National Institutes of Health. These studies were reviewed and approved by the Institutional Animal Care and Use Committee of Jilin University.

The murine macrophagocyte cell line RAW264.7 and human colorectal cancer cell line Caco‐2 were cultured in RPMI‐1640 medium supplemented with 10% fetal bovine serum (Biological Industries, BI), 100 units/mL of penicillin and 100 μg/mL of streptomycin (Medical Research Council, MRC) at 37°C with 5% CO_2_.

### 2.2 Bacterial strains, growth conditions and reagents

LM strain ATCC 19115 (serotype 4b) used in these studies was purchased from the American Type Culture Collection (ATCC). The bacteria were stored in sterile Trypticase (Tryptic) Soy Broth (TSB) medium containing 15% glycerol at −80°C. Frozen bacteria were streaked onto TSB agar and incubated at 37°C.

Baicalein used in this study was purchased from Sigma‐Aldrich (St.Louis, MO, USA). Baicalein was freshly prepared before each experiment. For animal assay, baicalein was suspended in 0.5% carboxymethylcellulose sodium (CMC) at a final concentration of 100 mg/kg. For other assays, baicalein was solubilized with dimethylsulfoxide (DMSO) to a storage density of 40 960 μg/mL.

### 2.3 Peptidase activity assay

The inhibitory effect of baicalein on LM sortase A (SrtA) was determined using a fluorescence resonance energy transfer (FRET) assay. Purified biologically active LM SrtA protein (stored at our lab) was incubated with different concentrations of baicalein in the reaction buffer (containing 50 mmol/L Tris‐HCl, 50 mmol/L CaCl_2_ and 150 mmol/L NaCl) for 30 minutes at 37°C in a black 96‐well plate before being triggered by the Dabcyl‐QALPTTGEE peptide substrate (Edans) (GL Biochem, Shanghai, China), followed by incubation for another hour. The changed peptidase activity of recombinant SrtA was measured at emission and excitation wavelengths of 350 and 520 nm, respectively, with a microplate reader. The value of the peptide substrate incubated with Proteinase K under the same conditions was used as a positive control.

### 2.4 Bacteria invasion analysis

For the invasion assay, Caco‐2 cells were seeded and grown in 24‐well‐plates at a density of 3×10^5^ per well and cultured overnight. ATCC 19115 were incubated with/without baicalein for 12 h with shaking (200 rpm) at 37°C. Bacteria were incubated with Caco‐2 cells at an MOI of 100:1 for 30 minutes with or without baicalein. The non‐adherent bacteria were moved by washing three times with PBS, and the remaining extracellular bacteria were killed with 20 μg/mL gentamicin (dissolved in RPMI‐1640 medium), followed by incubation for another 30 minutes. After three additional washes, the number of adherent and invasive bacteria was calculated by lysing Caco‐2 cells with sterile water and streaking them onto TSB agar plates.

### 2.5 Fluorescence labelling assay

Caco‐2 cells were infected with LM as described in the invasion analysis assay at an MOI of 100:1 with or without baicalein addition. At 1 h post‐infection (pi), infected Caco‐2 cells were washed three times with PBS, fixed with 4% paraformaldehyde for 20 minutes and incubated with blocking solution (5% BSA diluted in PBS) for 1 h. Extracellular bacteria were stained with LM‐specific antibody (Abcam) and secondary antibody conjugated to Alexa Fluor 488 (Invitrogen). Following permeabilization with 0.3% Triton X‐100 for 3 minutes, both intracellular and extracellular bacteria were stained with the same primary antibody and a secondary antibody conjugated to Alexa Fluor 594 (Molecular Probes). The images were captured using a confocal laser scanning microscope.

### 2.6 Haemolysis assay

Overnight ATCC 19115 cultures were expanded into fresh TSB at 1:100 with shaking for 2 h at 37°C, and then different concentrations of baicalein were added for further culture to an A600nm of 2.1. The supernatants were harvested by centrifugation (9 600 *g*, 2 min). Next, 200 μL supernatants or purified rLLO (20 ng/mL, stored in our lab) were first treated with different concentrations of baicalein at 37°C for 30 minutes in sterile acidic buffer (35 mmol/L Na_2_HPO_4_.12H_2_O, 0.125 mol/L NaCl, adjust pH value to 5.5 with acetic acid). Next, 2.5% rabbit erythrocytes (RBC) were added, followed by incubation for 15 minutes. The supernatant of the cocktail was harvested (10 000 rpm, 2 min) and measured. The A543nm of each sample was obtained after centrifugation (10 000 rpm, 1 min) to represent the haemolytic value. RBCs treated with ddH_2_O or acidic buffer served as a positive control or negative control, respectively. The percentage of lysed RBCs was determined by comparing each sample with the positive control, which was considered 100% hemolysis.

### 2.7 Bacterial growth curve

An overnight LM culture was expanded into fresh TSB at 1:100 with shaking for 3 h at 37°C. And then the indicated concentrations of baicalein were added for further culture at an A600nm of 0.1. The A600nm value of each sample was analysed hourly with an ultraviolet spectrophotometer. This work continued for 6 h until the A600nm stopped increasing.

### 2.8 Western blot assay

Bacterial culture was performed as described in the hemolysis assay. After the A600nm reached 2.1, equal volumes of supernatants of different samples were harvested by centrifugation (2 400 *g*, 5 minutes). Next, 5×SDS‐PAGE loading buffer containing β‐mercaptoethanol（β‐Me）was added, and the mixture was boiled for 10 minutes (100°C). Samples was separated by 12% SDS‐PAGE, and the proteins were transferred onto polyvinylidene fluoride（PVDF）membranes. After blocking the membranes in a blocking buffer (5% BSA) for 2 h at room temperature, the membranes were first incubated with primary rabbit antibody against LLO (Abcam, diluted with blocking buffer at 1:1000) and then a peroxidase‐conjugated secondary antibody (Proteintech, diluted with blocking buffer at 1:5000). The protein band signals were visualized with a Tanon‐4200 imager, using ECL reagent (Pierce™ ECL Western Blotting Substrate).

### 2.9 Oligomerization analysis assay

Recombined LLO protein was preincubated with or without the indicated concentrations of baicalein at 37°C for 30 minutes with acidic buffer and high concentrations of chloridion. In vitro oligomerization of LLO was detected by Western blot assay, as previously described.^8^, ^23^ The optical density of high weight oligomers and LLO monomers was analysed with ImageJ software.

### 2.10 Intracellular process analysis

RAW264.7 cells were seeded on 13‐mm coverslips in 24‐well‐plates and grown overnight before the assay. LM strain ATCC 19115 was cultivated in fresh sterile TSB overnight and added to cell medium at an MOI of 10 with/without baicalein for infection. Extracellular bacteria were removed and washed five times with PBS at 0.5 h post‐infection. The infected cells were then homogenized with sterile water or fresh RPMI‐1640 medium (containing 20 μg/mL gentamicin) for further incubation with or without baicalein. At 0.5, 2 and 5 h pi, by lysing cells, intracellular bacteria from each sample were released and counted by coating on TSB plates.

The infected RAW264.7 cells as described above were fixed with 4% paraformaldehyde for 30 minutes and then permeabilized with 0.3% TritonX‐100 at 0.5, 2 and 5 h pi Samples were then blocked with 5% BSA for 1 hour at room temperature following incubation with rabbit antibody against LM for 2 hours at room temperature. Bacteria were further incubated with Alexa Fluor 594‐conjugated secondary antibody (Molecular Probes) for 1 hour. F‐actin was stained with phalloidin coupled to Alexa 488 (Molecular Probes). The images were captured using a confocal laser scanning microscope.

For the bacterial Live/Dead assay, 5×10^6^ RAW264.7 cells were seeded into 100 mm dishes and grown overnight. The infection occurred as described above at an MOI of 10 with or without baicalein. Extracellular bacteria were killed at 0.5 h pi with 20 μg/mL gentamicin. At 5 h after infection, cells were washed with PBS and then lysed with 0.2% Triton X‐100 for 2 minutes. Survival of the intracellular bacteria was determined using a Live/Dead^®^ BacLightTM Bacterial Viability Kit (L13152).

### 2.11 Cell viability determination

RAW264.7 and Caco‐2 cells were seeded into 96‐well plates at 2×10^4^ cells/well at 37°C overnight before the assay. Bacteria were first cultured with or without baicalein treatment for at least 12 h. RAW264.7 cells were infected with LM without baicalein pretreatment at an MOI of 40, with or without baicalein addition while culturing. Caco‐2 cells were infected with LM with or without pretreatment at an MOI of 500, with indicated concentrations of baicalein. After 5 h of infection, lactate dehydrogenase（LDH）in culture supernatant was detected using a Cytotoxicity Detection Kit (LDH; Roche, Basel, Switzerland). The A492nm of each sample was measured, and the final percentage of dead cells was represented as (Sample‐Negative control)/( positive control–negative control). Cells with 0.2% Triton‐X100 and pure RPMI ‐1640 medium added were used as positive and negative controls, respectively. Infected RAW264.7 cells at 5 h pi were stained with live/dead reagent (Invitrogen) and photographed with a confocal laser scanning microscope (Olympus, Tokyo Japan).

### 2.12 Animal experiments

For animal experiments, overnight‐cultured ATCC 19115 was diluted 100‐fold in fresh TSB and grown at 37°C until reached an A600nm of 0.8. After gentle washing, bacteria were resuspended in PBS to the required concentration for the following assays. The mice were fasting for approximately 12 h before the assay. The mice were first treated with 200 μL bacterial suspension (containing 5×10^6^ CFU for mortality studies or 1×10^6^ CFU for bacteria load analysis) by intraperitoneal injection, and then administered 100 μL baicalein liquid orally every 12 h. Mice in the control group orally received 100 μL vehicle (0.5% CMC) at the same time after infection. Simultaneously, healthy mice were treated with 100 μL baicalein liquid in order to detect toxicity. Survival of the mice received lethal doses of LM was observed three times a day. At 60 h pi, mice infected with sublethal doses of LM were euthanized with anaesthesia following cervical dislocation. Then, the spleen and liver were homogenized, and the loaded bacterial numbers were counted by coating on TSB plates.

### 2.13 Statistical analysis

All experimental data were presented as the mean ± SEM (n ≥ 3). GraphPad Prism 5.0 was used for statistical analysis. The *P* values were determined through two‐tailed Student's *t* tests. Significance levels of *P* < 0.05 (*) and *P* < 0.01 (**) are indicated in the figures.

## 3 RESULTS

### 3.1 Baicalein incubation attenuates the biological activity of SrtA protein and bacterial internalization

**Figure 1:**
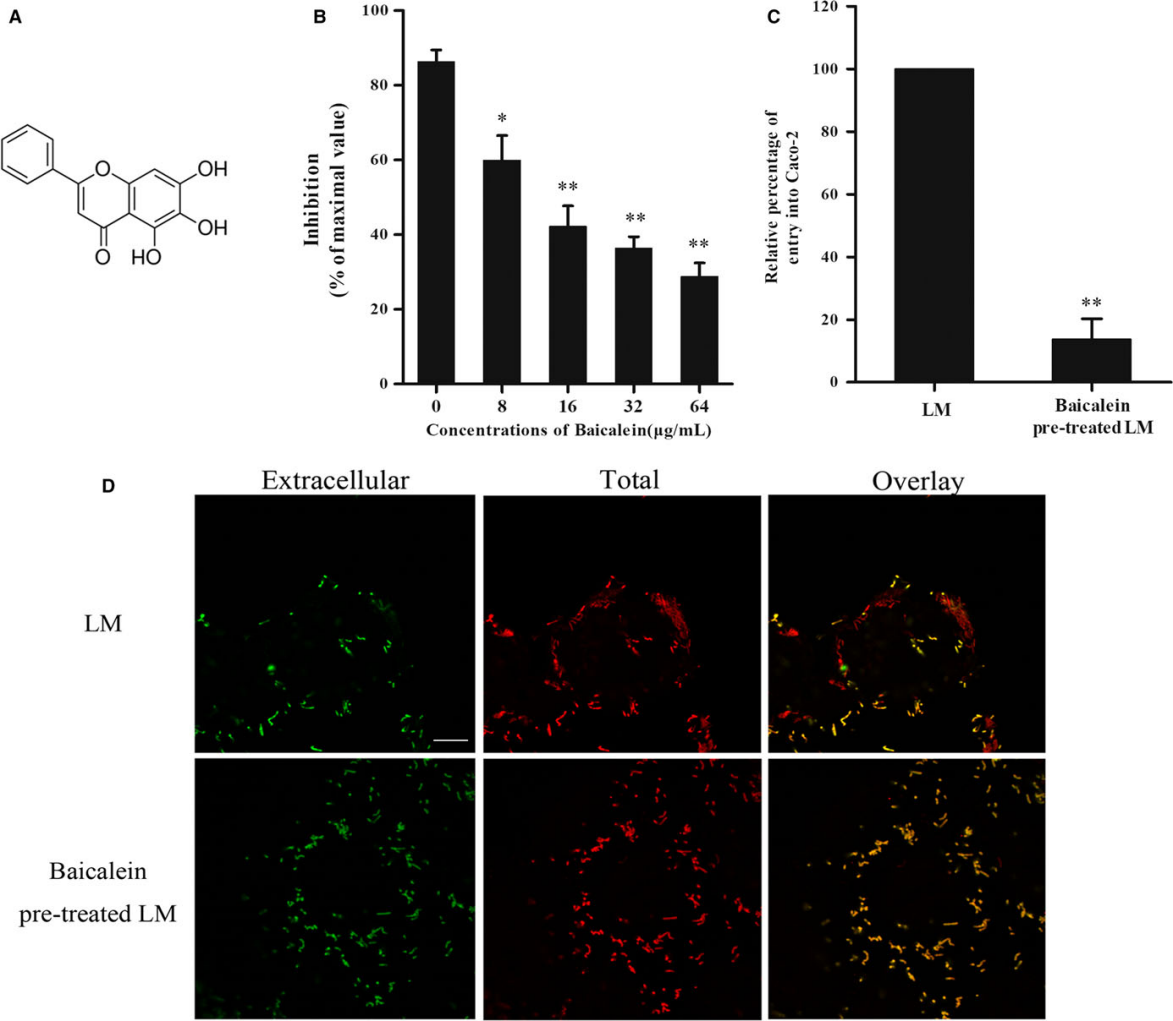
Baicalein inhibits listerial SrtA enzyme activity and interferes with the entrance of LM into Caco‐2 cells. A, Chemical structure of Baicalein. B, The inhibition of purified SrtA peptidase activity by baicalein. A FRET assay was employed to determine the inhibition rate of baicalein on purified SrtA in reaction buffer. The value of the reaction system with proteinase K was used as a positive control and maximal value. (C, D) The impact of baicalein on bacterial entry into Caco‐2 cells. LM was pretreated with or without baicalein overnight. C, An invasion assay was performed at an MOI of 100 for 1 h following CFU counting or electron microscopic observation. Cells infected with baicalein preincubated LM were compared with the control group. D, The extracellular LM was strained green with antibody‐conjugated Alexa Fluor 488, and the total bacteria were stained in red with antibody‐conjugated Alexa Fluor 594; scale bar = 10 μm. *, *P* < 0.05; **, *P* < 0.01

LM SrtA mediates surface protein anchoring onto peptidoglycan (PGN) of bacteria. SrtA specifically identifies and cleaves the LPXTG recognition motif sequence and mediates the transamidation reaction. The FRET assay was performed based on the peptidase activity of SrtA, and a flavonoid compound, baicalein (Figure 1A), was shown to significantly inhibit the activity of purified SrtA protein (Figure 1B) in a concentration‐dependent manner. To determine the potential influence of baicalein on LM internalization, an invasion assay was utilized. As expected, an obvious CFU decrease was observed in human intestinal Caco‐2 cells infected with baicalein pretreated LM compared to cells infected with LM without preincubation (Figure 1C). After 1 hour of infection, extracellular and intracellular bacteria were visualized with immunofluorescence staining (Figure 1D). In the infected control group (without baicalein incubation), the minority of LM were stained both red and green, whereas most of the LM were stained with only red fluorescence, which indicated that the majority of cell‐associated bacteria were intracellular. Consistent with the result of the invasion assay, samples infected with baicalein‐pretreated LM were observed to have opposite staining, wherein the minority of cell‐associated bacteria was stained red. Consequently, these results suggest that baicalein is an LM SrtA inhibitor that efficiently attenuated LM internalization.

### 3.2 Baicalein strongly inhibits LLO pore‐forming activity by targeting oligomerization

**Figure 2:**
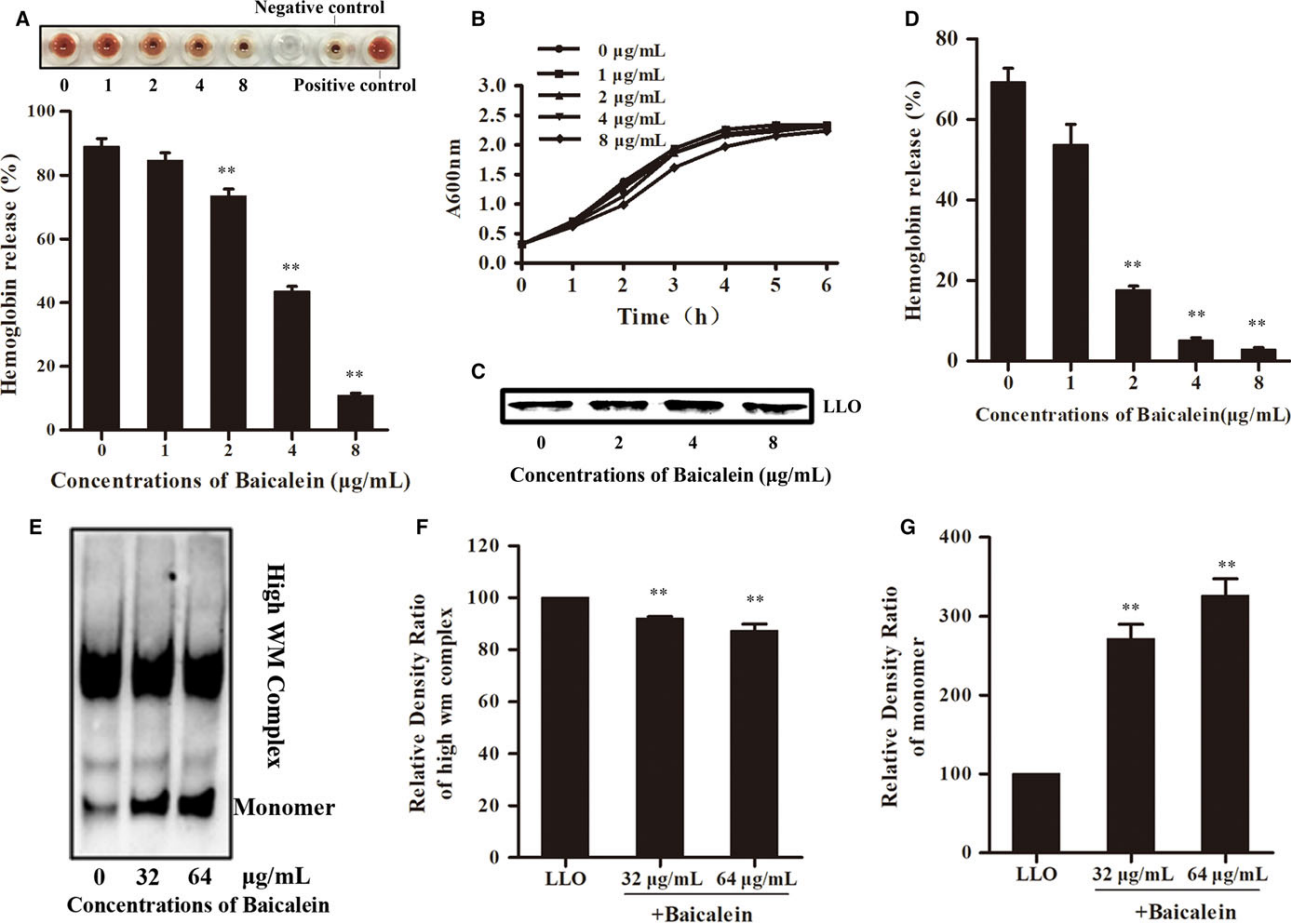
Baicalein inhibits the pore‐forming activity of listeriolysin O (LLO) without the inhibition of bacterial growth and LLO production. A, The higher picture shows a visual image. The lower picture shows the inhibition of haemolytic activity of the bacterial culture supernatant by the indicated concentrations of baicalein. B, The growth curve of LM co‐cultured with various concentrations of baicalein. C, Western blotting analysis of LLO expression in culture supernatant. Bacteria were cultivated as described above. The same volume of culture supernatant was harvested at an A600nm of 2.1 and treated as described in Western blot assay. D, The hemolysis assay was performed with purified LLO protein, as described, in an acidic buffer. E, The impact of Baicalein on LLO oligomerization visualized with a Western blot image. F and G, The optical density of LLO oligomers and monomers analyzed with ImageJ software. **, *P* < 0.01

The LM hemolysin, LLO, plays a special role in LM pathogenicity. To determine if baicalein targeted LLO, a haemolysis assay was performed to detect the effect of baicalein on the pore‐forming activity of secreted LLO. Baicalein‐incubated bacterial culture supernatant had significantly reduced the hemolytic activity, which occurred in a dose‐dependent manner (Figure 2A). Bacterial growth was detected to investigate whether the decreased hemolytic activity was associated with the inhibition of bacterial survival (Figure 2B). The results indicated that active concentrations on LLO hemolytic activity showed no significant influence on bacteria growth. A Western blot assay was then performed to investigate whether the effect of baicalein on LLO was due to an influence on LLO production. The LLO content in the LM culture supernatant was not significantly different between the baicalein‐treated group and control group (Figure 2C), which suggested that Baicalein may directly target LLO. Then, we performed a hemolysis assay with purified LLO protein. Baicalein incubated with LLO attenuated hemolytic activity (Figure 2D). In addition, we performed an oligomerization assay to visual LLO oligomers and monomers by Western blot assays (Figure 2E). When analysing the bands of oligomers and monomers, the baicalein‐treated samples had increased monomers and decreased oligomers, indicating a reduced oligomerization (Figure 2F,G). Hence, the results demonstrated that baicalein significantly inhibited LLO hemolytic activity in vitro, and this effect is due to an inhibition of the LLO oligomerization process.

### 3.3 Baicalein inhibits the escape of bacteria from the phagophore to the cytoplasm

**Figure 3:**
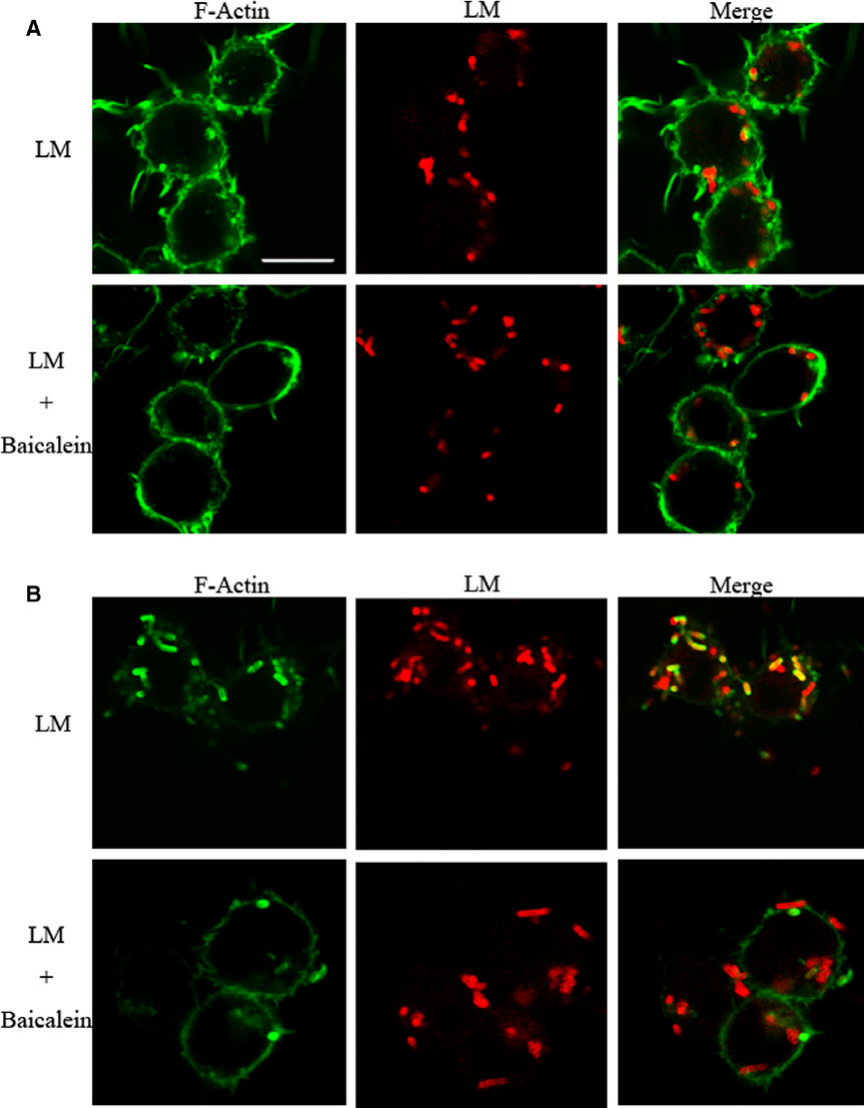
The influence of Baicalein on intracellular process of LM at 0.5 and 2 h post‐infection (pi) The images show bacterial (red) and F‐actin (green) recruitment. RAW264.7 cells were infected with LM at an MOI of 10. Following washing and fixing, macrophages were blocked with 5% BSA and then labeled by immunostaining with specific antibodies at 0.5, 2 and 5 h pi. The images were captured using a confocal laser scanning microscope. Scale bar, 10 μm. A, Bacteria and F‐actin staining at 0.5 h post‐infection. B, Bacteria and F‐actin staining at 2 h post‐infection

**Figure 4:**
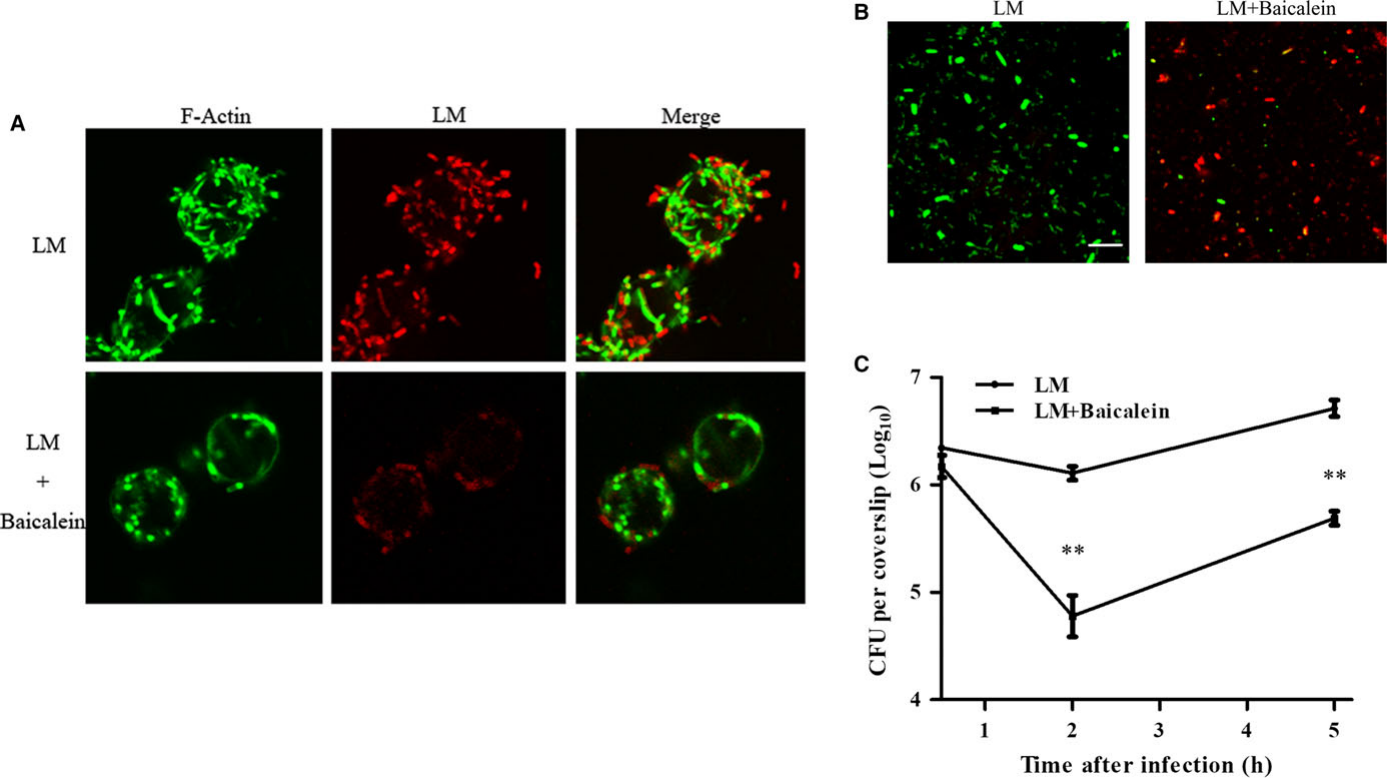
Baicalein influence on intracellular bacteria at 5 h pi (A) Bacteria and F‐actin staining at 5 h after infection. B, Influence of baicalein on intracellular bacterial viability. Infection was performed as described. The lysates of infected cells at 5 h pi were stained with Syto 9 (live bacteria stain green) or propidium iodide (dead bacteria stain red); scale bar = 10 μm. C, The inhibition of baicalein on bacterial growth in RAW264.7 cells. Infected cells were lysed with 0.2% Triton‐X100 at 0.5, 2 and 5 h after infection. Intracellular bacteria were analyzed with a CFU assay. **, *P* < 0.01

Phagosome escape is required for bacteria to evade being broken down by hydrolytic enzymes secreted into vacuoles. The bacteria may exploit a host system of actin‐based motility to move within cells and into neighbouring cells in an LLO‐mediated way. To investigate the effect of baicalein on the intracellular processes of LM, bacteria and F‐actin in infected RAW264.7 cells were immunostained in red or green at 0.5, 2, and 5 h (Figure 4A) post‐infection, respectively. LM in infected cells with baicalein incubation showed delayed and decreased actin assembly at 2 h post‐infection (Figure 3B), when LM in cells without baicalein incubation already showed actin assembly at 0.5 h post‐infection (Figure 3A), which led to decreased bacterial escape. Therefore, baicalein incubation inhibits the escape of LM from vacuoles. This effect may be mainly related to the inhibition of LLO.

### 3.4 Baicalein addition attenuates bacterial growth rate and facilitates the clearance of LM

To demonstrate the influence of baicalein on the intracellular processes of LM, all cell‐associated bacteria at 0.5, 2, and 5 h pi were counted using a CFU assay. The CFU burden in baicalein co‐incubated cells was not significantly different from non‐treated infected cells at 0.5 h pi, but significantly decreased at 2 and 5 h pi (Figure 4C). The results suggested that baicalein addition slowed down the replication of LM in RAW264.7 cells. Bacteria in infected cells at 5 h post‐infection were harvested by lysing cells, and then, live and dead bacteria were stained in green or red, respectively. Compared with the control group, the infected cells cotreated with baicalein contained more dead bacteria (Figure 4B), indicating that baicalein addition facilitated bacterial clearance in macrophages. Altogether, baicalein inhibited bacterial growth and contributed to the elimination of bacteria.

### 3.5 Baicalein incubation inhibits LM‐induced cell injury

**Figure 5:**
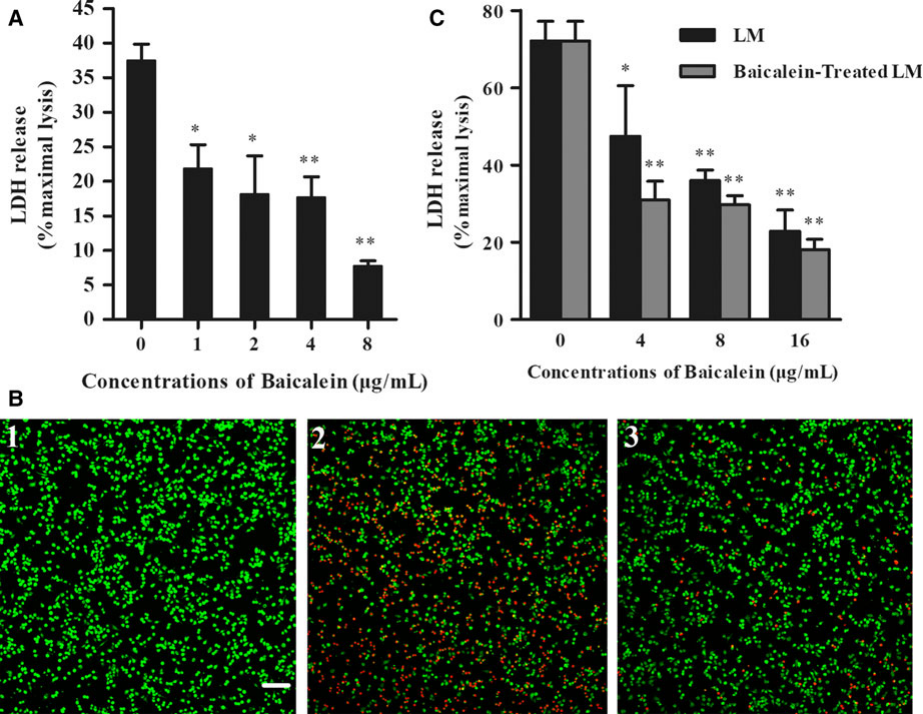
Baicalein addition decreased LM‐induced cellular injury in a dose‐dependent manner. A, The effect of baicalein on LM‐infected RAW264.7 cells. Released relative lactate dehydrogenase (LDH) in the coculture system was determined with a cytotoxicity detection kit at 5 h pi (B) Live/Dead cells staining. Cells were stained with the Live/Dead (green/red) reagent at 5 h pi and then observed under a confocal laser scanning microscope. (B1) Uninfected cells (Negative control); (B2) RAW264.7 cells infected with LM; (B3) Cells co‐cultured with LM and 8 μg/mL baicalein. C, The effect of baicalein on LM‐infected Caco‐2 cells LDH release at 5 h pi. *, *P* < 0.05; **, *P* < 0.01.

Based on the previous study, we assumed that baicalein may protect cells from LM infection. An LDH release assay was performed at a high MOI (40 to RAW264.7 cells and 500 to Caco‐2 cells) to detect protection against infection. After 5 h of infection, released LDH in RAW264.7 cells (Figure 5A) and Caco‐2 cells (Figure 5C) supernatant without baicalein coculture showed an obvious increase compared to the negative control group, while both Caco‐2 cells and RAW264.7 cells showed insusceptibility to the indicated concentration of baicalein (data not shown). Consistent with our expectation, the detected LDH in the baicalein‐treated samples significantly decreased in a concentration‐dependent way. A live/dead cell staining assay was performed at the same time (5 h after infection). Live cells were stained in green, and dead cells were stained in red (Figure 5B). The visual images were consistent with the LDH assay. Furthermore, preincubated LM showed less pathogenicity in Caco‐2 cells than bacteria without baicalein pretreatment (Figure 5C). Together, baicalein efficiently protected cells from LM‐induced injury.

### 3.6 Baicalein protects mice from LM infection

**Figure 6:**
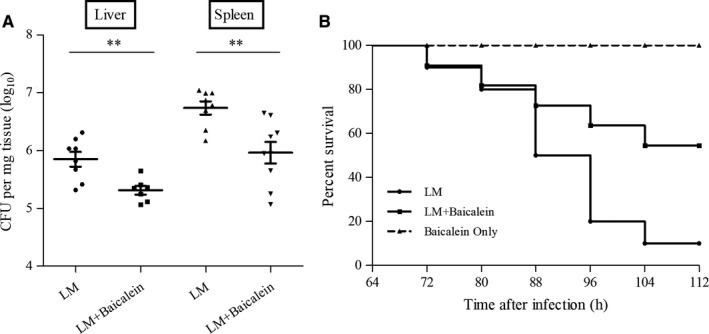
Baicalein protects mice from LM infection. 6‐8‐week‐old mice were infected with LM intraperitoneally and orally treated twice a day with baicalein or with CMC as a control. A, Analysis of bacterial burdens in the liver and spleen. The mice were infected with sublethal doses of bacteria and were mercy‐killed at 60 h pi (B) Survival analysis of infected or uninfected mice treated with baicalein. Mice were infected with lethal doses of bacteria and were observed every 8 h. **, *P* < 0.01

We further investigated whether baicalein could have a therapeutic effect on combating LM infection in an animal model. At 60 h post‐infection, compared with the control group, significant decreases of bacteria burdens were shown in the spleens and livers of baicalein‐treated mice receiving a sub‐lethal dose of LM (Figure 6A). In addition, at 112 h post‐infection, 90.00% of animals that received a lethal dose of bacteria were dead, and 54.55% of baicalein‐treated infected mice survived (Figure 6B). Moreover, mice that only received baicalein were survived without visible damage until the end of the experiment, which means that indicated concentrations of baicalein showed no significant toxicity in mice. Taken together, baicalein treatment obviously protected LM‐infected mice from death.

**Figure 7:**
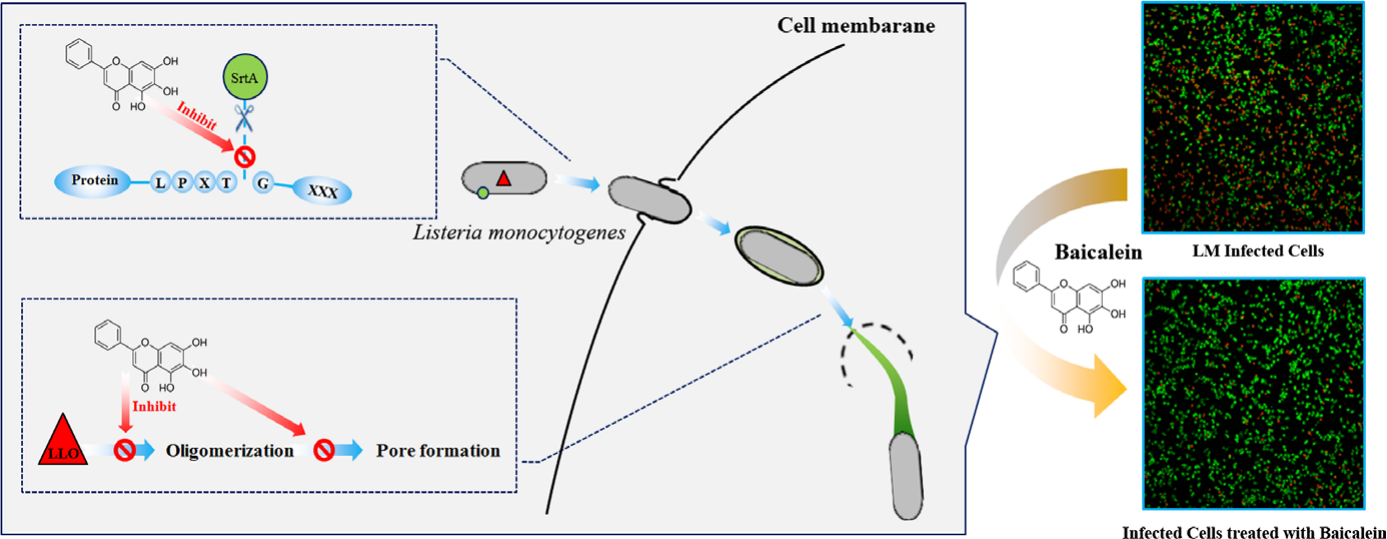
A graphical abstract expresses the inhibition of baicalein of LM infection process

## 4 DISCUSSION AND CONCLUSIONS

High concentrations of antibiotics are recommended for the treatment of LM. However, the excessive antibiotics use raises a series of health considerations and accelerates antimicrobial resistance. Previous studies have demonstrated that the inhibition of virulence factors with several small molecule compounds significantly decreases the pathogenicity of LM and may slow the emergence of multidrug resistance.^8^, ^24^ Moreover, many highly conserved sequences or structures were identified in some virulence factors of gram‐positive bacteria, such as LM, *Staphylococcus aureus*, *Streptococcus pneumonia*, and *Streptococcus suis*, which means that the strategy against listerial virulence factor may be extended to other pathogens.^25^ Baicalein has been reported to be an inhibitor of *Staphylococcus aureu*s biofilm formation, and we assumed that baicalein can also influence listerial virulence.^26^

In this study, baicalein was determined to efficiently block listerial sortase A (SrtA) cleavage activity and listeriolysin O (LLO) haemolytic activity with a pre‐incubation in two absolutely distinct assays, which suggested a potential influence of baicalein towards proteins, such as changing secondary structure, or covalently binding in active site region residues, rather than inhibition on virulence expression, bacterial growth or substrates of the reactions.^6^, ^27^ It differed from the direct suppression on bacterial survival by traditional antibiotics or targeting only one virulence factor, might due to a synergistic effect on therapy against LM or other gram‐positive pathogen infection.

The number of cell‐associated bacteria was similar between baicalein cotreated cells and non‐treated cells at 0.5 h after infection in assays performed with RAW264.7 cells. However, in the invasion assay performed with Caco‐2 cells, the number of intracellular bacteria in cells infected with baicalein‐pretreated LM was significantly less than in cells with non‐treated LM at 1 h after infection. The defective cellular internalization in the invasion assay may be due to the longer baicalein treatment of LM, which induced more influence on SrtA. In fact, longer treatment of LM with baicalein indeed attenuated infected mammalian cells injury, which obtained an excellent agreement with our speculation and confirmed the therapeutic potential of baicalein in vitro. Besides, the deferred vacuole escape and suppressed proliferation of LM in baicalein co‐cultured macrophage cells suggested that baicalein may also aid in bacterial clearance by host innate immunity.

Baicalein is an acknowledged flavonoid with multiple biological activities. Studies have suggested that baicalein was more suitable for oral administration in rat owing to the well and rapid absorption in stomach and small intestine.^28^ In our study, baicalein was given to LM‐infected mice with an oral dose of 200 mg/kg body weight in one day, significantly reduced bacterial colonization in livers and spleens, and protected mice from death with the dose showed no sign of toxicity. In fact, baicalein has been proved to be a safe agent with maximum tolerated dose of 15 400 mg/kg in mice, and be tolerated with single oral doses of 100‐2800 mg by human subjects.^29^, ^30^

In conclusion, baicalein significantly inhibited bacterial colonization and multiplication by targeting both listerial virulence factor SrtA and LLO (Figure 7), attenuated LM‐induced infection both in vitro and in vivo at an innoxious dose. All the results together demonstrated that baicalein might be a safe and potential efficient therapeutic for listeriosis.

## CONFLICT OF INTEREST

The authors have no conflict of interest to declare.
